# Comparison of exploratory behavior of male and female woodlice (*Armadillidium vulgare*)

**DOI:** 10.1590/1414-431X2023e12902

**Published:** 2023-10-20

**Authors:** M. Cazentine, R. Bonuti, S. Morato

**Affiliations:** 1 Laboratório de Comportamento Exploratório, Faculdade de Filosofia, Ciências e Letras de Ribeirão Preto, Universidade de São Paulo, Ribeirão Preto, SP, Brasil

**Keywords:** Exploratory behavior, Terrestrial isopods, Armadillidium vulgare, Sex differences, Open-field test, Dry/moist box

## Abstract

There are several studies in the literature showing that male and female rats explore novel environments and exhibit different exploration patterns when submitted to different apparatuses. In general, female rats spend more time moving and exploring the apparatuses than males do. A previous study showed that male woodlice (*Armadillidium vulgare*) explore novel environments in a very similar way to male rats (*Rattus norvegicus*) when tested in apparatuses analogous to the open-field test and light/dark box. Since that study was conducted only with male rats and woodlice, and since they exhibited very similar patterns of behavior, the present experiment aimed at investigating whether male and female woodlice explore novel environments with different behavioral patterns. Female and male woodlice were tested in the open-field and in the dry/moist box. Results obtained in the open-field test showed that both males and females remained longer in the corners than along the walls and avoided staying in the center. However, females remained longer along the walls and less in the corners. In the dry/moist box, there were no significant differences between the sexes: both females and males remained significantly longer in the moist compartment.

## Introduction

Living beings in nature exhibit peculiar behaviors to deal with a variety of situations. Most exhibit exploratory behaviors in their environment in order to find food, mating partners, a place to live or hide, to avoid exposure to predators, or to discover new assets in the environment. Different species may exhibit behavioral patterns that differ in the details but are analogous in the results. Although it is possible to investigate exploratory behavior in nature, it is frequently studied in laboratory settings, with rodents being most frequently studied (1-11). Exploratory behavior has also been studied in a great variety of animals, such as ferrets (12), domestic cats (13-15), domestic dogs (15,16), gerbils (17), four species of fish (18), or even terrestrial isopods (19). In these studies, there seem to be no differences in exploratory behavior between females and males. In rats, however, females seem to explore novel environments in a somewhat different way than males (10,20-34).

In a recent paper, Bonuti et al. (19) showed that male woodlice explore novel environments in a similar way to rats: when submitted to a square open-field apparatus, both species explore the novel environment by moving more frequently along the walls, remaining much longer in the corners, and avoiding the central area. In the light/dark box or its woodlice counterpart, the dry/moist box, both species remain longer in the preferred area: dark for the rats, moist for the woodlice. However, this study comparing rats and woodlice did not include females. This raises the question of whether female and male woodlice exhibit different patterns of behavior when exploring new environments.

Thus, the present experiment is part of a broader study aimed at using woodlice to determine the effects of drugs on exploratory behavior in different apparatuses. The goal of the present study was to compare the exploratory behavior of male and female woodlice in the open-field test and in the dry/moist box.

## Experiment 1: Comparing exploratory behavior of male and female woodlice in an open-field

On the one hand, male rats and woodlice explore an open-field with the same behavior patterns (19); on the other hand, male and female rats show different patterns of exploratory behavior (10,20-34). Thus, it would be interesting to study whether male and female woodlice also exhibit different patterns of exploratory behavior. The present experiment aimed to investigate the behavior of male and female woodlice submitted to an open-field test.

## Material and Methods

### Subjects

Forty woodlice (*Armadillidium vulgare*) of both sexes (20 males and 20 females) weighing approximately 50 mg were used. The woodlice were captured at the campus of the University of São Paulo in Ribeirão Preto, Brazil, and males were separated from the females. They were kept in a polypropylene box (40×25×7 cm) with a 2-cm layer of washed sand (quartz sand No. 00, Yellowfish Ecommerce, Brazil) with two wood blocks (10×5×1.5 cm) under which the isopods could hide. A 500-mL plastic bottle filled with water with a 3-mm hole in the bottom provided a continuous flow of water to maintain the humidity of the sand substrate. The isopods were fed TetraFin Goldfish Flakes (Tetra Holding, USA) and tortoise commercial chow (Alcon, Brazil) *ad libitum*. The food was replaced every three days. The animal room was maintained in a 12-h light/dark cycle (lights on at 7:00 a.m.) with the temperature kept between 24 and 27°C.

All testing was performed between 7:30 and 11:30 a.m. Although not mandatory, all experimental procedures were carried out in accordance with the guidelines of the Brazilian Society for Neuroscience and Behavior for animal care and with the American Psychological Association (APA) ethical guidelines.

### Apparatus

A square black Plexiglas open-field (10×10×5 cm) was used. The floor of the apparatus was lined with a 10×10 cm square of common white copy paper (75 g/m^2^). In order to avoid eventual pheromone effects, the paper lining was replaced after testing each woodlice. Illumination was provided by a white 4-W LED lamp placed 15 cm above the floor, which yielded around 500 lux.

### Procedure

All behavioral tests were recorded by a video camera placed above the apparatus and connected to a video recorder. The videos were subsequently analyzed and behaviors were scored with the X-PloRat software (35). To record where the behaviors occurred, the open-field floor image was divided into twenty-five 2-cm squares by an open source software for video recording (OBS Studio, https://obsproject.com/).

Subjects of both sexes were tested individually. The males were recorded on one day and the females on the next day. Each subject was gently placed in the center of the apparatus and allowed to freely explore for 10 min. The behaviors recorded were: time spent in the areas (center, near the walls, and in the corners) and the number of squares crossed.

### Data analysis

Data were analyzed with the SigmaStat software (Systat Software Inc., USA). Data are reported as means±SE and analyzed by a two-way analysis of variance (ANOVA) followed, whenever appropriate, by the Tukey test. In all cases a significance level of P<0.05 was used.

## Results

**Figure 1:**
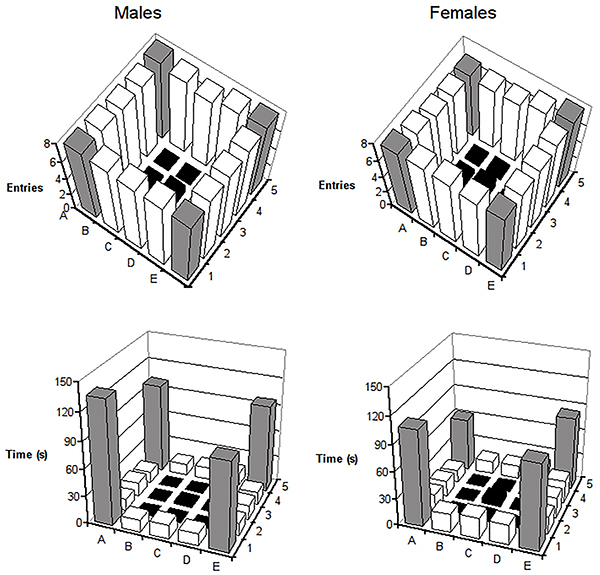
Mean number of entries into each square (upper panel) and mean time spent in them (lower panel) by males and females. Black columns indicate squares in the center of the open-field, light gray columns indicate squares in the corners, and white columns indicate squares close to the walls.

The upper part of Figure 1 shows the average entries into each of the squares by both males and females. A similar pattern of exploration by the two sexes was seen. The number of entries into the squares showed a strong preference for moving close to the walls. However, the lower part of Figure 1 shows that subjects from both sexes tended to remain longer in the corners and very little time in the center of the apparatus.

**Figure 2:**
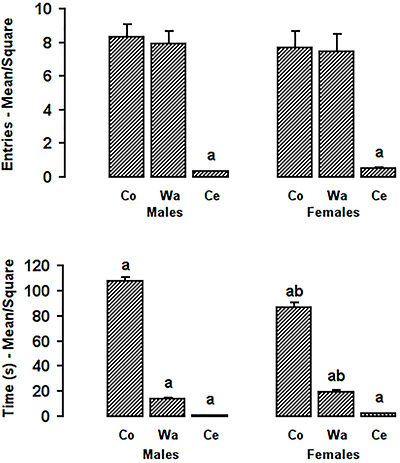
Mean number of entries (upper panel) and mean time (lower panel) spent in the areas of the open-field by male and female woodlice. Co: Corners; Wa: Walls; Ce: Center. Data are reported as mean and SE. ^a^P<0.05 compared with the two other areas. ^b^P<0.05 compared with the corresponding area of males (Tukey test).

Figure 2 (upper part) shows the average entries into each square of each area of the open-field by males and females. ANOVA did not show a major effect of sex in frequency of entries (F[1,114]=0.22, P=0.64), but it showed a major effect of area (F[1,114]=66.61, P<0.01) and no interaction between factors (F[2,114]=0.16, P=0.85). The Tukey test showed that subjects of both sexes entered the squares in the center significantly less than the squares in the corners and along the walls (P<0.01).

The time spent in the different areas of the open-field is shown in the lower part of Figure 2. ANOVA showed significant differences between sexes (F[1,114]=7.56, P<0.01), areas (F[2,114]=1363.24, P<0.01), and an interaction between sex and area (F[2,114]=25.410, P<0.01). The Tukey test showed that both males (P<0.01) and females (P<0.01) spent more time in the corners than along the walls and more time along the walls than in the center. However, the Tukey test showed that females remained in the corners significantly less time than males (P<0.01), and they remained longer along the walls than males (P=0.04). The test showed no differences in the time spent in the center (P=0.60).

## Discussion

The results showed that males did not differ from females in locomotion pattern. Subjects of both sexes visited the squares along the walls equally, including the four corners, more than the squares in the center. Also, animals of both sexes avoided remaining in the squares in the center of the apparatus. Male woodlice remained a little longer than females in the corners than in the squares in the other areas. On the other hand, compared with males, females remained longer in the squares along the walls. These two results may indicate a tendency of females being more explorative.

## Experiment 2: Comparing exploratory behavior in a two-environment apparatus with different degrees of aversiveness

The present experiment aimed to compare the exploratory behavior of male and female woodlice in a two-environment apparatus: the dry/moist box (19). Since woodlice tend to avoid dry places and approach moist ones (36), this apparatus contrasts a dry compartment with a humid one (37).

## Material and Methods

### Subjects

Twenty male and 20 female woodlice (*Armadillidium vulgare*, approximately 50 mg) were used. The subjects had similar characteristics as described in Experiment 1.

### Apparatus

A transparent 10×5×5 cm Plexiglas box was used. A 5×5 cm piece of common white copy paper (75 g/m^2^) was placed on one side of the apparatus and humidified with 10 drops of water (the moist compartment). Another 5×5 cm piece of the same white copy paper was placed without water on the other side (the dry compartment). The woodlice could move freely from one piece of paper to the other.

### Procedure

All behavioral tests were recorded by a video camera placed above the apparatuses and connected to a video recorder. The videos were subsequently analyzed and behaviors were scored with the X-PloRat software (35). To record where the woodlice were, the apparatus floor image was divided into two 5×5 cm squares by an open source software for video recording (OBS Studio, https://obsproject.com/). We recorded the number of crossings from one compartment to the other and the total time spent in each compartment. Subjects of both sexes were tested individually. Since we were also interested in avoidance behavior, each woodlice was gently placed in the center of the moist compartment and allowed to freely explore for 10 min.

### Data analysis

Data are reported as means±SE. The time spent in each of the two areas was analyzed by a two-way ANOVA (sex: male × female and area: dry × moist) followed, whenever appropriate, by the Tukey test. The frequency of transitions from one compartment to the other was analyzed by the Student *t*-test. In all cases a significance level of P<0.05 was used.

## Results

**Figure 3:**
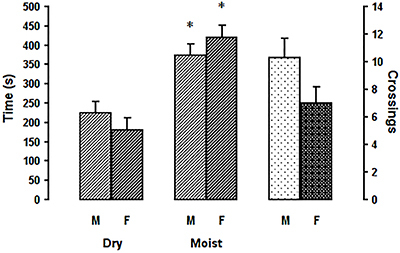
Time spent in the 2 compartments of the dry/moist box by male and female woodlice and frequency of crossings from one side to the other. M: males; F: females. Data are reported as mean and SE. *P<0.05 compared to the dry compartment (Tukey test).

Figure 3 shows the time spent in each compartment. Subjects of both sexes explored the apparatus in a similar way. Females and males remained longer in the moist area (P<0.001), and females tended to cross from one side to the other less than the males (right side of the figure), although without statistical significance (t(38)=1.824, P=0.076). ANOVA showed major effects of area (F[1,76]=39.47, P<0.01), but not sex (F[1,76]<0.001, P=1.00), nor sex and area interaction, (F[1,76]=2.06, P=0.155).

## Discussion

Results showed no significant differences between male and female woodlice: subjects of both sexes remained longer in the moist area than in the dry area and there was no statistical difference in the frequency of crossings. This indicated that both sexes of animals of this species equally preferred moist environments rather than dry ones, as previously suggested (18,36-38), which contrasts with previous studies that found some differences between sexes in exploratory behavior of rats (10,20-26,32,34,39,40).

## Conclusions

In general, the exploratory behavior of male woodlice was very similar to that of females in terms of motor activity. Concerning time allocation in areas, the only difference observed in Experiment 1 involved less time in the corners and walls but no difference in occupation of the central area. Experiment 2 showed no significant differences between the sexes in the occupation of the spaces. Thus, female and male woodlice did not exhibit different patterns of exploratory behavior in these two apparatuses.
